# Disease Burden at the Time of Transplantation Is a Primary Predictor of Outcomes in Pediatric MDS: A Single-Center Experience

**DOI:** 10.3390/cancers17101645

**Published:** 2025-05-13

**Authors:** Ann Dahlberg, Phil Stevenson, Neel S. Bhatt, Lauri Burroughs, Paul A. Carpenter, Corinne Summers, Katherine Tarlock, Monica S. Thakar, Filippo Milano, H. Joachim Deeg, Marie Bleakley

**Affiliations:** 1 Clinical Research Division, Fred Hutch Cancer Center, Seattle, WA 98109, USA; pstevens@fredhutch.org (P.S.); nbhatt@fredhutch.org (N.S.B.); lburroug@fredhutch.org (L.B.); pcarpent@fredhutch.org (P.A.C.); corinne.summers@seattlechildrens.org (C.S.); katherine.tarlock@seattlechildrens.org (K.T.); msthakar@fredhutch.org (M.S.T.); fmilano@fredhutch.org (F.M.); jdeeg@fredhutch.org (H.J.D.); mbleakle@fredhutch.org (M.B.); 2 School of Medicine, University of Washington, Seattle, WA 98195, USA; 3 Seattle Children’s Hospital, Seattle, WA 98105, USA; 4 Translational Sciences and Therapeutics Division, Fred Hutch Cancer Center, Seattle, WA 98109, USA

**Keywords:** myelodysplastic syndrome, pediatric, hematopoietic cell transplant, outcomes

## Abstract

Background: Hematopoietic cell transplantation (HCT) remains the only curative therapy for pediatric myelodysplastic syndrome (MDS) in all but rare cases. While HCT outcomes for pediatric MDS are similar across the largest registry and single-center trials, factors identified as contributing to inferior outcomes vary from study to study. We performed an analysis to provide more clarity on the prognostic implications of disease characteristics, including blast burden and cytogenetic abnormalities, in the current era. Methods: We conducted a retrospective analysis of 36 consecutive children (<18 years of age at HCT) who underwent allogeneic HCT for MDS between June 2000 and October 2019 at the Fred Hutchinson Cancer Center. Results: Overall survival (OS) was 77% (95% CI 64–92%) and relapse-free survival (RFS) was 71% (95% CI 57–88%) at 2 years post-HCT. Patients with <5% blasts by morphology in the bone marrow at the time of HCT showed superior 2-year OS at 87% (95% CI 74–100%) as compared to 54% (95% CI 32–93%) in patients with ≥5% blasts, consistent with an HR of 4.6 (CI 1.14–18.7, *p* = 0.03). The inferior outcomes in patients with ≥5% blasts were due to increased relapse incidence (HR 7.6, CI 1.5–39.3) with no difference in NRM or acute GVHD. Conclusions: OS and RFS were comparable to what has been observed in other large, single-center studies (OS 77%, RFS 71% at 2 years) and compared favorably to outcomes from the largest multi-center retrospective analyses.

## Simple Summary

Myelodysplastic syndrome is a rare and serious blood disease in children that can lead to leukemia. A stem cell transplant is often the only chance for a cure, but it is difficult to predict which children will respond well to this treatment. This study analyzed 36 patients who received transplants at a single center to better understand how disease features affect outcomes. Children with fewer abnormal cells, referred to as blasts, in their bone marrow at the time of transplant had significantly improved survival rates. Chemotherapy given before transplant helped lower disease levels but did not always prevent relapse, especially in patients with more aggressive disease. These high-risk patients had a two-year survival rate of just over 50%, compared to over nearly 90% in other children. This suggests that current treatments may not be sufficient to overcome aggressive disease. More effective and less toxic approaches are urgently needed, both before and after transplant, to improve outcomes. Developing targeted therapies and understanding the unique genetic characteristics in pediatric patients will be key to making progress. This study highlights the importance of continuing research to help more children survive, particularly those with the highest risk disease.

## 1. Introduction

Myelodysplastic syndrome (MDS) accounts for less than 5% of all pediatric hematologic malignancies, with an incidence of 1–4 cases per million per year [1,2]. Akin to MDS in adults, it is a clonal disorder of hematopoiesis associated with cytopenias and has a significant risk of transformation to acute myeloid leukemia (AML). However, morphologic, cytogenetic, and molecular findings generally differ between children and adults, and pediatric MDS is more frequently associated with inherited bone marrow failure syndromes (IBMFSs) [3]. Consensus for the classification of pediatric MDS has improved over time, with most patients now classified under the WHO 2016 criteria, although the 2022 criteria that further integrate genomic characteristics have recently been added [4,5,6]. Despite these guidelines, the relative rarity of the diagnosis and its heterogenous presentation pose challenges for determining disease outcomes.

Hematopoietic cell transplantation (HCT) remains the only curative therapy for pediatric MDS in all but rare cases [7]. Unlike in adult patients, non-HCT therapies such as immunosuppression or hypomethylating agents have shown limited and often transient efficacy in children, making timely HCT essential for long-term survival [8]. HCT accessibility and outcomes have improved over time for patients without an HLA-matched sibling or unrelated donor. The largest registry trials show approximately 60% overall survival (OS) following HLA-matched donor HCT. Non-relapse mortality (NRM) and disease recurrence contribute equally as primary causes of treatment failure [9,10]. Single-institution trials have reported similar or more favorable OS, NRM, and disease recurrence results across stem cell sources, with the exception of lower relapse rates reported more recently in a single-center pediatric cohort who received umbilical cord blood transplantation or αβT- and B-cell-depleted HLA-haploidentical transplantation [11,12,13,14,15].

While HCT outcomes for pediatric MDS are similar across the largest registry and single-center trials [11,12,13,14,15], factors identified as contributing to inferior outcomes vary from study to study. HLA-mismatched unrelated donor (MMUD) HCT, excluding cord blood and haploidentical donor sources, resulted in inferior outcomes related to increased incidence of relapse or NRM [9,10]. High-risk disease characteristics such as high myeloblast count at the time of HCT and the type or complexity of cytogenetics have also been variably associated with outcome. Similarly, the time from diagnosis to HCT and the administration of pre-HCT chemotherapy are inconsistently associated with OS and relapse-free survival (RFS). Reported differences in outcomes are likely due to the heterogeneity of pediatric MDS and low patient numbers. In addition, many reports include patient cohorts spanning several decades, while transplant strategies have evolved. Two recent single-center analyses showed clear survival improvement for more recently transplanted children with MDS [12,13]. Therefore, we performed an updated analysis to provide more clarity on the prognostic implications of disease characteristics, including blast burden and cytogenetic abnormalities, in the current era. The analysis includes results of 36 consecutive children transplanted for MDS between 2000 and 2019 at the Fred Hutchinson Cancer Center with standardized supportive care and GVHD management guidelines.

## 2. Materials and Methods

### 2.1. Patients

We conducted a retrospective analysis of 36 consecutive children (<18 years of age at HCT) who underwent allogeneic HCT for MDS between June 2000 and October 2019 at the Fred Hutchinson Cancer Center. All patients were transplanted on institutional review board-approved treatment protocols or standard treatment plans. All patients, guardians, or both provided written informed consent to the institutional review board-approved protocol for the collection of data for analysis.

### 2.2. Donor Selection

Stem cell sources included HLA-matched sibling donor (MSD) bone marrow or peripheral blood stem cells (PBSCs), HLA-matched or mismatched unrelated donor (MUD or MMUD) bone marrow or PBSCs, or unrelated cord blood (CB). For recipients of MSD, MUD, or MMUD, allele-level typing was performed at HLA-A, -B, -C, -DRB1, and -DQB1. For recipients of CB, antigen typing was performed at HLA-A and -B and allele-level typing at HLA-DRB1.

### 2.3. Supportive Care

All patients were hospitalized at Seattle Children’s Hospital in single rooms, ventilated with high-efficiency particulate air filtration. Prophylactic broad-spectrum antibiotics were administered to all patients during the neutropenic period. All patients received *Pneumocystis jirovecii* prophylaxis preceding HCT and then restarted approximately 28 days after HCT through at least 6 months post-HCT. Anti-fungal prophylaxis with fluconazole was given to all patients unless prior fungal history necessitated escalated therapy. Patients with serology-proven prior herpes simplex or varicella zoster virus exposure received prophylactic acyclovir. Recipients of CB with positive serology for cytomegalovirus (CMV) pre-HCT received high-dose acyclovir. Intensive CMV monitoring occurred in all patients post-HCT. Filgrastim was administered to recipients of CB beginning on day +1 until an absolute neutrophil count (ANC) > 2500/µL for 3 consecutive days.

### 2.4. Statistical Methods

Neutrophil engraftment was defined as the 1st of 2 consecutive days of an absolute neutrophil count ANC > 500/µL. Acute and chronic GVHD was diagnosed and graded clinically per established criteria [16,17]. OS was defined as the time from HCT (day 0) to death or last contact. Relapse was defined as a morphologic or cytogenetic recurrence of disease post-HCT. RFS was defined as the time from HCT (day 0) to relapse, death, or last contact. NRM was defined as death after HCT for any reason other than relapse.

Descriptive statistics were used to summarize patient and disease characteristics. Univariate Cox models were used to test associations of potential risk factors with OS, relapse, NRM, and acute GVHD grades II–IV and III–IV. Kaplan–Meier or cumulative incidence plots were constructed for those risk factors with significant associations (i.e., *p* < 0.05). All computations and plots were created using R version 4.2.2.

## 3. Results

### 3.1. Patient and Disease Characteristics

Patient, disease, and HCT characteristics are shown in Table 1. The median age at HCT was 11.7 (range: 1–18) years. The median time of follow-up was nearly 3 years (0.2–15.2 years). Fifteen patients (41.7%) had primary MDS, six (16.7%) had a diagnosis of prior severe aplastic anemia, eight (22.2%) had a known underlying MDS-predisposing gene mutation, and seven (19.2%) developed MDS following therapy for a prior malignancy or HCT. Patients with juvenile myelomonocytic leukemia, Fanconi-anemia, or MDS who had progressed to acute myelogenous leukemia (defined as a blast count ≥ 20%) were excluded. Patients were retrospectively scored using the WHO 2016 MDS classification for pediatrics and categorized into RCC (refractory cytopenia of childhood), MDS-EB1 (MDS with excess blasts 1), MDS-EB2 (MDS with excess blasts 2), and TA-MDS (therapy-associated MDS) [4]. Cytogenetics were normal in six patients (16%). Monosomy 7/del 7q was the most common chromosomal abnormality and was detected in 18 patients (50%). Molecular testing was performed in 10 patients (Table 2; see disease details). The median time from diagnosis to HCT was 126 (range: 21–1981) days.

### 3.2. HCT Characteristics and Engraftment

Donor sources were evenly split between MSD, MUD, MMUD, and CB. The majority were conditioned with busulfan and cyclophosphamide (*n* = 21, 58%), while six patients (16.7%) received high-dose total body irradiation (TBI)-based conditioning. The remaining nine patients received reduced toxicity conditioning; in eight, this was treosulfan-based [18,19]. The median time to ANC engraftment was 17 (range: 11–33) days. One recipient of an MMUD graft had primary graft failure and died from early TRM following the 2nd transplant.

### 3.3. Overall and Relapse-Free Survival

**Figure 1:**
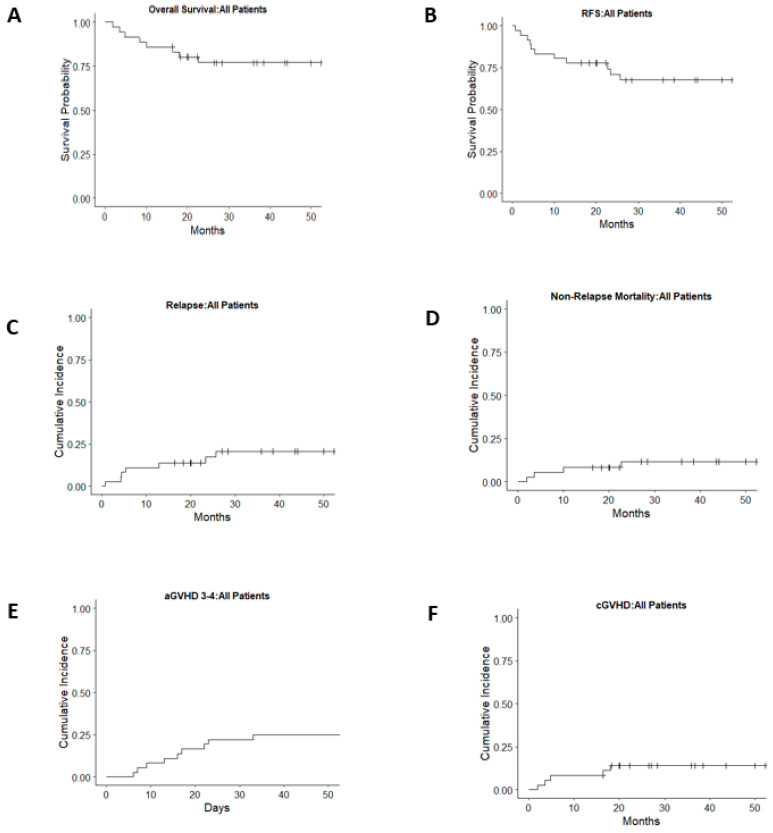
Overall survival (**A**), relapse-free survival (**B**), relapse (**C**), non-relapse mortality (**D**), acute GVHD grades III–IV (**E**), and chronic GVHD (**F**) for all patients.

**Figure 2:**
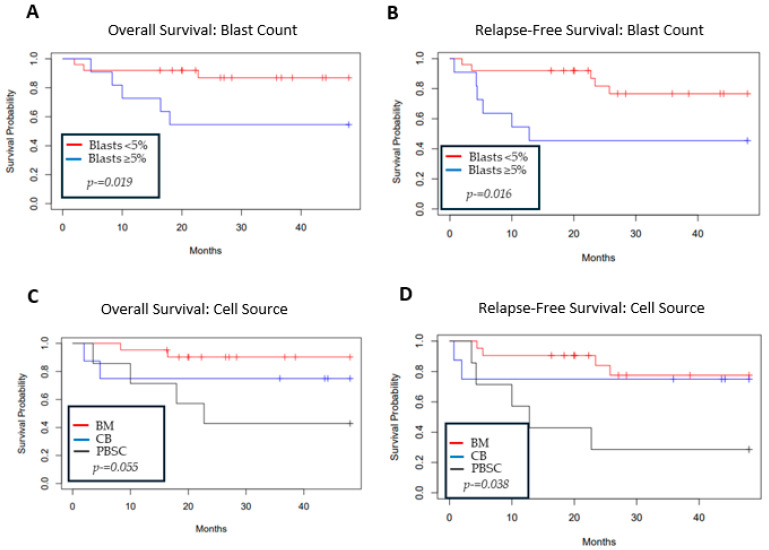
Overall survival and relapse-free survival for blast count before transplant (**A**,**B**) and transplant cell source (**C**,**D**). “No-5%” refers to blast count < 5%; “5%” refers to blast count ≥ 5%.

The OS was 77% (95% CI 64–92%) (Figure 1A) and the RFS was 71% (95% CI 57–88%) at 2 years post-HCT (Figure 1B). Patients with <5% blasts by morphology in the bone marrow at the time of HCT showed superior 2-year OS at 87% (95% CI 74–100%) as compared to 54% (95% CI 32–93%) in patients with ≥5% blasts, consistent with an HR of 4.6 (CI 1.14–18.7, *p* = 0.03) (Figure 2A, Table 3). The inferior outcomes in patients with ≥ 5% blasts were due to increased relapse incidence (HR 7.6, CI 1.5–39.3), with no difference in NRM or acute GVHD (Table 3). As a result, RFS in patients with ≥5% blasts was also reduced to 45% (95% CI 24–87%) compared to 83% (95% CI 68–99%) in those with <5% blasts (Figure 2B). Patients transplanted with PBSCs as compared to cord blood or bone marrow (BM) had an inferior OS and RFS but no difference in NRM (Figure 2C,D, Table 3). No other factors, including age, conditioning intensity, sibling vs. unrelated donor, presence of monosomy 7, karyotype complexity, WHO 2016 MDS classification, time from diagnosis to HCT, administration of chemotherapy pre-HCT, or year of HCT, impacted OS, although the latter neared significance when dichotomized by decade or treated as a continuous variable (Table 3). Given the infrequent events in this patient population, multivariate analysis was not feasible. In addition to a higher myeloblast count at the time of HCT, RFS was also worse with the administration of chemotherapy pre-HCT. Five patients received pre-transplant chemotherapy (Table 2) with a reduction in blast count from >5% at diagnosis to ≤5% at the time of transplant. Despite this, RFS was 77% (95% CI 63–94%) in patients who did not receive chemotherapy compared to 30% (95% CI, 6–100%) in those who did receive chemotherapy. This corresponded to an HR of 4.9 (95% CI 1.4–17, *p* = 0.01, Table 3).

### 3.4. Relapse

There were seven relapses after HCT for MDS (Table 2). One patient achieved a second remission following the taper of immune suppression. Three patients underwent a second HCT with subsequent relapse or early NRM, resulting in death. Two patients relapsed with AML following the first HCT and remained alive after the second HCT (for AML). One patient had a very early post-HCT relapse and died without pursuing additional therapy. The two-year estimate of relapse was 17% (95% CI 8–36%) (Figure 1C). Factors associated with increased relapse risk included a blast count ≥ 5% (Figure 2B) (HR 7.6, 95% CI 1.47–39.3, *p* = 0.016) and the administration of chemotherapy pre-HCT (HR 7, 95% CI 1.53–31.9, *p* = 0.012). No other factors were associated with increased relapse (Table 3).

### 3.5. GVHD

Day-100 estimates of grades II-IV and grades III-IV acute GVHD were 75% (95% CI 56–86%) and 25% (95% CI 10–38%, Figure 1E), respectively. The 2-year incidence of chronic GVHD was 28% (95% CI, 10–42%, Figure 1F). The presence of either grade II-IV or grade III-IV acute GVHD did not significantly impact OS or relapse. There was an increased risk of NRM in patients with acute grade III-IV GVHD (HR 9.7, 95% CI 1.05–89.5, *p* = 0.04). Of the 36 patients included, 7 received PBSC grafts (1 MSD, 1 MUD, and 5 MMUD). PBSC recipients accounted for four of the nine cases of grade III–IV acute GVHD, including one of the two cases of grade IV GVHD observed. Due to the limited number of PBSC recipients, formal statistical analysis was not performed.

### 3.6. Non-Relapse Morbidity and Mortality

NRM at 2 years post-HCT was 12% (95% CI 5–30%) (Figure 1D). No factors other than acute GVHD grades III-IV were associated with an increased risk of NRM. There was a trend towards decreased risk of NRM when time was treated as a continuous variable (HR 0.77, 95% CI 0.58–1.02, *p* = 0.07); however, when dichotomized by decade, this trend was not observed. NRM was not increased in patients who received chemotherapy pre-HCT (HR 2.48, 95% CI 0.25–24.2, *p* = 0.43).

Twenty-seven patients survived until the last follow-up. Causes of death were infection (n = 2), GVHD (n = 3), and relapse (n = 4). HCT was generally well tolerated, with a single death occurring in the first 100 days post-HCT due to grade IV acute GVHD. The median time from the date of HCT to hospital discharge was 29 (range: 17–68) days. Seven patients required intensive care unit (ICU)-level care during their first hospitalization, including four who developed sinusoidal obstructive syndrome (SOS). Post-HCT infections in the first 100 days included 11 patients with bloodstream infections, 10 with CMV reactivation, 3 with EBV viremia, 5 with BK viruria, 4 with RSV, and 5 with presumed fungal pneumonia.

## 4. Conclusions

In this single-center, retrospective study, we report HCT outcomes of a contemporary cohort of consecutive children with MDS. OS and RFS were comparable to what has been observed in other large, single-center studies (OS 77%, RFS 71% at 2 years) and compared favorably to outcomes from the largest multi-center retrospective analyses. Results do not appear to differ from our center’s experience in the prior 2 decades (1976–2001) when OS for MDS (excluding JMML) was 68–74% based on MDS classification at the time [20]. In the present cohort, the primary disease factors that correlated with inferior OS and/or RFS and relapses were a higher disease burden at the time of HCT and the administration of chemotherapy pre-HCT. All patients who received pre-HCT chemotherapy demonstrated a reduction in blast count to ≤5% at the time of transplantation; however, their risk of relapse remained high. This may be due to inherent disease characteristics that are not overcome with current chemotherapy or transplant approaches and not due to the pre-HCT chemotherapy itself. While molecular profiling was not available for all patients and statistical analysis was not possible, based on the limited profiling data available, patients who received pre-HCT chemotherapy had complex molecular profiles consistent with a baseline high-risk disease state. Numbers were insufficient to analyze the subpopulation of patients with blast burden > 5% to determine whether pre-HCT chemotherapy impacted outcomes in this group. A larger patient cohort will be required to address this question. It is not clear why patients who received PBSC had inferior OS and RFS compared to patients given other stem cells, despite no difference in NRM. It is possible that there was a bias in that PBSCs were selected for patients with a higher concern for relapse risk to exert a greater graft-versus-leukemia effect. While descriptive data suggest a possible association between PBSC grafts and higher rates of severe acute GVHD in our cohort, the number of PBSC recipients was too small to allow for meaningful statistical analysis. In addition, MMUDs were overrepresented in the PBSC graft recipients, further complicating the analysis of an association between PBSCs and severe acute GVHD. Larger studies will be necessary to better evaluate the relationship between graft source and acute GVHD risk in pediatric MDS.

We tested a multitude of other factors that have previously been reported to be associated with post-HCT outcomes, including age at HCT, time to HCT, WHO 2016 MDS classification, karyotype complexity, and the presence of monosomy 7. None of these was associated with an inferior prognosis. Of note, monosomy 7 was examined in this study given its established association with poor prognosis in adult MDS and in some earlier pediatric reports; however, more recent data suggest that in pediatric MDS, monosomy 7 alone is not predictive of inferior outcomes unless accompanied by additional complex structural abnormalities [8,21,22]. There was a trend towards improved OS in patients transplanted more recently, and this appeared to be due to decreased NRM and not relapse. Our data suggest that efforts focused on lowering disease burden before HCT may be most impactful on post-HCT outcomes. However, intensive chemotherapy approaches appear inadequate.

While it is generally accepted that patients with MDS should undergo HCT, the decision of whether to debulk the disease pre-HCT in higher-risk patients often depends upon the treating physician’s opinion. Nakano et al. recently surveyed 28 large HCT centers regarding their approach to MDS and reported considerable variability with a higher probability of cytoreductive therapy (either hypomethylating agent (HMA) or intensive chemotherapy) being given to patients with higher blast counts [23]. Approximately 20% of centers report pursuing cytoreductive therapy with blast counts at 5–9%, while over half would pursue cytoreductive therapy with blast counts at 10–15%. The choice of cytoreductive therapy remains challenging, as results to date are mixed regarding the impact of pre-HCT therapy, with data from earlier studies with intensive chemotherapy showing higher NRM. It is also important to note that patients with idiopathic bone marrow failure syndromes are at higher risk of toxicity from intensive chemotherapy [24]. While patients in our study with a lower blast burden had improved outcomes, we were not able to determine whether an intervention with pre-HCT chemotherapy in those with higher blast counts was able to improve outcomes. Large meta-analyses in adults, preceding the use of venetoclax, demonstrate no significant impact on post-HCT outcomes, while smaller pediatric studies suggest improved outcomes in patients with high-risk disease who received pre-HCT HMA [25,26,27,28]. Further, prior MDS analyses have shown a possible advantage of intensive chemotherapy preceding HCT in the highest-risk patients [27]. Studies in both adults and pediatric MDS suggest that venetoclax-based regimens can be used as an effective bridge to HCT and, in combination with HMAs, may offer a less toxic alternative to intensive chemotherapy [29,30,31]. Prospective trials are needed to identify optimal pre-HCT therapeutic agents and strategies for pediatric MDS. In addition to pre-HCT interventions aimed at lowering disease burden, post-HCT relapse prevention strategies are being pursued for high-risk patients. Small retrospective analyses suggest improved outcomes in patients who receive post-HCT hypomethylating agents with or without additional interventions such as donor lymphocyte infusion (DLI) [32,33,34]. Our center has adopted a strategy of reducing the blast burden to <5% pre-HCT in patients with lower-intensity approaches, including HMAs, as well as pursuing post-HCT HMA therapy for relapse prevention. Prospective trials of post-HCT therapies to reduce relapses are needed in patients at high risk of relapse.

Our study also showed no difference in outcomes between patients receiving high-intensity myeloablative conditioning (most commonly busulfan and cyclophosphamide) versus those receiving a reduced toxicity conditioning with treosulfan, fludarabine, and low-dose TBI [18,19,35]. Reduced toxicity conditioning approaches may be of particular interest in patients who have MDS due to underlying inherited marrow failure syndromes such as GATA2 deficiency that increase the risk for infection, or patients with treatment-associated MDS and a history of prior intensive chemotherapy.

Major drawbacks of our study include its small cohort size and its retrospective nature. While our cohort is similar in size to other single-center analyses, the limited patient number and relatively few relapses prevented meaningful multivariate analysis. Retrospective registry studies include greater patient numbers but introduce significant heterogeneity in supportive care as well as diagnostic testing for MDS. Single-center analyses, therefore, remain an important addition to our understanding of HCT in pediatric MDS. Another important limitation is the lack of comprehensive molecular and genetic testing across the cohort. Although molecular data were available for 10 patients, these were insufficient for meaningful subset analysis. Furthermore, routine screening for inherited bone marrow failure syndromes, including telomeropathies and SAMD9/SAMD9L mutations, has only become standard practice more recently. As a result, some underlying germline predispositions may have been undetected in this historical cohort. Additionally, while we tested multiple factors for association with outcome, the relatively small cohort size and low number of adverse events precluded meaningful multivariate analysis.

Overall, we observed favorable HCT outcomes in children with MDS. Although the rarity of pediatric MDS limits the size of most single-center studies, including ours, the consistency of our findings with prior reports underscores the value of this independent, uniformly treated cohort in contributing to the broader understanding of disease and transplant-related risk factors. In line with the previously published literature, we identified blast counts ≥ 5% and the receipt of pre-HCT chemotherapy as the only significant factors associated with inferior post-HCT outcomes. For these high-risk patients, 2-year OS was 54%, compared to 80% in the remaining patients. Future investigations aimed at this population with interventions to control disease burden pre-HCT, particularly targeted agents and those with less toxicity, may be needed to improve overall outcomes. Additionally, future studies should further elucidate the prognostic and therapeutic implications of genomic alterations commonly seen in pediatric MDS, including mutations in RAS pathway genes, RUNX1, SETBP1, and ASXL1, which differ significantly from those observed in adult populations. An improved understanding of these genetic distinctions will be critical for refining risk stratification and developing targeted therapies uniquely suited to pediatric patients.

## Figures and Tables

**Table 1:** Patient and disease characteristics.

Characteristic	*n*
Gender	
Male	17
Female	19
Median Age	11.7 years (1–18)
Type MDS	
Primary, no underlying cause	15
Evolved from SAA	6
Secondary (from malignancy or prior intensive chemotherapy)	7 (3 ALL, 1 Burkitt, 1 sarcoma, 1 NB, 1 SCID s/p first transplant) (20%)
Underlying predisposition *	8
Classification by the WHO 2016 criteria	
RCC	17
MDS-EB1	8
MDS-EB2	4
TA-MDS	7
Cytogenetics	
Monosomy 7/Del 7q	18
Trisomy 8	7
Complex	4
Del 5	1
Normal	6
Pre-HCT Therapy	
Chemotherapy for MDS (not prior malignancy)	5
IST	6
None	25
Time from diagnosis to HCT	
<4 months	15 (median 89 days)
≥4 months	21 (median 162 days)
Year of HCT	
2000–2008	19
2009–2019	17
Pre-HCT blast count	
<5%	25
≥5%	11
Graft Source	
MSD (BM)	8
MSD (PBSC)	1
MUD (BM)	10
MUD (PBSC)	1
MMUD (BM)	3
MMUD (PBSC)	5
CBT	8
Conditioning Regimen	
Busulfan/Cyclophosphamide +/− ATG	21
TBI/Cyclophosphamide	6
Reduced toxicity	8
Other	1
Graft failure	
Yes	1 (2nd HCT early TRM)

* Underlying predisposition syndromes: familial MDS (1), SAMD9 (2), ELA2/Kostmann (1), MPL mutation (2), GATA2 germline (1), RUNX1 germline (1).

**Table 2:** Disease details.

	Age	Measurable Disease at Transplant (% Blasts)	WHO-2016 Classification at Time of Diagnosis	Donor Source	Conditioning	Time to Transplant (Days)	Pre-tx Chemo	Relapse (Days)	Cause of Death	Disease Detail
**1**	10.8	7	RCC	CBT	HD-TBI/Cy	134			Infection (CMV)	Prior history of SAA (received IST); Monosomy 7
**2**	16.1	0	RCC	MUD BMT	Bu/Cy	366				Prior history of SAA (received IST); Monosomy 7
**3**	15.7	4	TA-MDS	MSD BMT	Bu/Cy	104				TA-MDS (sarcoma) Inversion 6, t(11;19)
**4**	3	0	RCC	CBT	HD-TBI/Cy/ATG	60			GVHD	Prior history of SAA (received IST); Normal karyotype
**5**	3.5	4.6	TA-MDS	MSD BMT	Bu/Cy	110				TA-MDS (neuroblastoma) Trisomy 8
**6**	11.7	5	RCC	MUD BMT	Bu/Cy	165				Familial amegakaryocytic dysplasia Monosomy 7
**7**	13	3	RCC	MUD PBSC	Bu/Cy	28			GVHD/infection	Prior history of SAA (received IST); Del 7q- on one marrow
**8**	15.6	>5%	MDS-EB1	MUD PBSC	Bu/Cy	336			Chronic GVHD	Prior history of SAA (received IST); Normal karyotype
**9**	14.1	5	MDS-EB1	MSD BMT	Bu/Cy/ATG	114	ADE × 2	132	Relapse *	Normal karyotype
**10**	12.8	7	TA-MDS	MSD BMT	Bu/Cy/ATG	63		132	Relapse **	TA-MDS (ALL) Complex karyotype
**11**	14.3	13	MDS-EB2	MSD PBSC	Bu/Cy/ATG	134		384		Post-relapse reemission after IST taper Del-7q (1; 7)
**12**	1.8	3	TA-MDS	CBT	HD-TBI/Cy/ATG	162				TA-MDS (Burkitt)
**13**	1	1.5	RCC	MUD PBSC	HD-TBI/Cy	126				Monosomy 7
**14**	11.1	0	RCC	MUD BMT	Bu/Cy/ATG	971				Familial amegakaryocytic dysplasia Normal karyotype
**15**	13.6	2.8	RCC	MUD PBSC	Bu/Cy	121				Prior history of SAA (received IST); Monosomy 7
**16**	12.1	5	MDS-EB1	MUD PBSC	Bu/Cy	131		128	Relapse **	Trisomy 8
**17**	17.9	1	RCC	MUD PBSC	Flu, TBI (3 Gy)	1981	Etop/Dex (Received for secondary HLH and not MDS)		Respiratory failure/infection ***	Graft failure after 1st transplant; early TRM after 2nd transplant; Trisomy 8
**18**	6.8	10	MDS-EB2	MUD BMT	HD-TBI/Cy	153				Monosomy 7
**19**	10.4	1	RCC	MUD BMT	Bu/Cy	142				Familial marrow failure; Complex karyotype
**20**	11.6	0	TA-MDS	MUD BMT	Bu/Cy	84				TA-MDS (ALL); Del (18)
**21**	16.8	5	MDS-EB2	CBT	Treo/Flu/TBI (2 Gy)	168				Trisomy 8
**22**	6.8	1.6	RCC	MSD BMT	Treo/Flu/TBI (2 Gy)	77				Monosomy 7
**23**	2.2	1	RCC	MUD BMT	Bu/Cy	186				SAMD9, familial monosomy 7
**24**	15.2	1	RCC	CBT	Treo/Flu/TBI (2 Gy)	614				Familial thrombocytopenia; RUNX1 heterozygous; Del 5q
**25**	12.1	6	MDS-EB1	CBT	Treo/Flu/TBI (2 Gy)	119				ELA-2 mutation (received chronic G-CSF) Monosomy 7
**26**	2.8	2	RCC	MSD BMT	Treo/Flu/TBI (2 Gy)	84				Monosomy 7
**27**	17.6	4	MDS-EB1	CBT	Treo/Flu/TBI (2 Gy)	94				NGS-positive GATA2 (confirmed germline); Monosomy 7
**28**	8	0	MDS-EB2	MSD BMT	Treo/Flu/TBI (2 Gy)	133	ADE	772 ****		Monosomy 7, NGS-positive CSF3R, NF1, PTPN11
**29**	6.9	0	RCC	MSD BMT	Bu/Cy	124				Germline GATA-2 Monosomy 7
**30**	3.2	2	TA-MDS	MUD BMT	Bu/Cy	115				TA-MDS (SCID/Omenn syndrome with MDS s/p 1st transplant) Complex karyotype
**31**	1.8	0	RCC	MUD BMT	Bu/Cy	191				SAMD9, familial monosomy 7, NGS otherwise negative
**32**	1.5	5	MDS-EB1	CBT	Treo/Flu/TBI (2 Gy)	94		21	Relapse/MOF	Monosomy 7, NGS-positive NRAS
**33**	13.7	1	RCC	MUD BMT	Bu/Cy	98				Monosomy 7, NGS-negative
**34**	13	1	MDS-EB2	MUD BMT	HD-TBI/Cy	184	Decitabine followed by mito/Ara-C			NGS-positive PTPN11, TET2 Trisomy 8
**35**	9.5	1	MDS-EB1	MUD BMT	Bu/Cy	82	Decitabine and ruxolitinib	702 ****		NGS-positive for JAK2, TP53 Trisomy 8
**36**	12	0	TA-MDS	MUD BMT	Bu/Cy	126				TA-MDS (ALL) 2nd transplant (1st transplant for ALL) Complex karyotype

* Relapse after first transplant, subsequent transplant with relapse. ** Underwent second transplant and died with early TRM. *** Graft failure after first transplant, early TRM, after 2nd transplant. **** Relapse with AML after first transplant, remains in remission following 2nd cord blood transplant.

**Table 3:** Hazard ratios of variables impacting OS, relapse incidence, NRM, and RFS.

	Hazard Ratio *	95% CI	*p*-Value
Overall Survival			
Age (≤12 years vs. >12 years)	1.63	0.44–6.1	0.47
Donor source (MSD vs. MUD/MMUD/CBT)	1	0–∞	1
Stem cell source (BM vs. CB; BM vs. PBSC)	4.39; 6.29	0.7–26.3; 1.14–34.57	0.1; 0.03
Pre-transplant chemotherapy (No therapy vs. therapy)	2.43	0.49–12.13	0.28
Monosomy 7 (No vs. Yes)	0.46	0.12–1.85	0.27
Complex karyotype (<3 abnormalities vs. ≥3)	0.51	0.06–4.07	0.52
Blast percent at transplant (<5% vs. ≥5%)	4.62	1.14–18.7	0.03
Time from dx to transplant (<120 days vs. ≥120 days)	0.47	0.12–1.77	0.26
Conditioning intensity (MAC vs. RIC)	1	0.2–4.87	0.99
Year of transplant (2000–2008 vs. 2009–2019)	0.14	0.02–1.18	0.07
Relapse			
Age (≤12 years vs. >12 years)	2.03	0.45–9.16	0.36
Donor source (MSD vs. MUD/MMUD/CBT)	0.79	0.08–7.22	0.83
Stem cell source (BM vs. CB; BM vs. PBSC)	0.71; 2.2	0.07–6.38; 0.4–12.19	0.76; 0.36
Pre-transplant chemotherapy (No therapy vs. therapy)	6.72	1.47–30.7	0.014
Monosomy 7 (No vs. Yes)	0.64	0.14–2.88	0.56
Complex karyotype (<3 abnormalities vs. ≥3)	1.7	0.38–8.84	0.53
Blast percent at transplant (<5% vs. ≥5%)	7.23	1.39–37.46	0.02
Time from dx to transplant (<120 days vs. ≥120 days)	0.46	0.1–2.08	0.32
Conditioning intensity (MAC vs. RTC)	1.24	0.24–6.42	0.8
Year of transplant (2000–2008 vs. 2009–2019)	0.75	0.17–3.35	0.7
Non-Relapse Mortality			
Age (≤12 years vs. >12 years)	0.94	0.15–5.69	0.94
Donor source (MSD vs. MUD/MMUD/CBT)	1.03	0–∞	1
Stem cell source (BM vs. CB; BM vs. PBSC)	Too few events to calculate		
Pre-transplant chemotherapy (No therapy vs. therapy)	2.48	0.25–24.2	0.43
Monosomy 7 (No vs. Yes)	0.21	0.02–1.87	0.16
Complex karyotype (<3 abnormalities vs. ≥3)	Too few events to calculate		
Blast percent at transplant (<5% vs. ≥5%)	1.62	0.26–9.98	0.6
Time from dx to transplant (<120 days vs. ≥120 days)	0.81	0.13–4.96	0.82
Conditioning intensity (MAC vs. RIC)	0.92	0.1–8.35	0.94
Year of transplant (2000–2008 vs. 2009–2019)	1	1	0.99
Relapse-Free Survival			
Age (<12 vs. ≥12)	1.47	0.47–4.61	0.5
Donor source (MSD vs. MUD/MMUD/CBT)	0.78	0.09–7.1	0.82
Stem cell source (BM vs. CB; BM vs. PBSC)	2.13; 4.89	0.48–9.55; 1.3–18.37	0.32; 0.02
Pre-transplant chemotherapy (No therapy vs. therapy)	4.77	1.38–16.5	0.013
Monosomy 7 (No vs. Yes)	0.42	0.13–1.42	0.16
Complex karyotype (<3 abnormalities vs. ≥3)	0.9	0.19–4.12	0.89
Blast percent at transplant (<5% vs. ≥5%)	3.8	1.19–12.09	0.02
Time from dx to transplant (<120 days vs. ≥120 days)	0.58	0.19–1.82	0.35
Conditioning intensity (MAC vs. RIC)	1.11	0.3–4.12	0.88
Year of transplant (2000–2008 vs. 2009–2019)	0.37	0.1–1.4	0.14

* Hazard ratio expresses outcome in the second term relative to the first.

## Data Availability

De-identified data available upon request due to patient privacy concerns.

## Abbreviations

**Abbreviation**	**Full Term**
HCT	Hematopoietic cell transplantation
MDS	Myelodysplastic syndrome
OS	Overall survival
RFS	Relapse-free survival
HR	Hazard ratio
NRM	Non-relapse mortality
GVHD	Graft-versus-host disease
AML	Acute myelogenous leukemia
IBMFS	Idiopathic bone marrow failure syndrome
MMUD	Mismatched unrelated donor
MSD	Matched sibling donor
PBSC	Peripheral blood stem cell
MUD	Matched unrelated donor
CB	Unrelated cord blood
CMV	Cytomegalovirus
TBI	Total body irradiation
ANC	Absolute neutrophil count
ICU	Intensive care unit
SOS	Sinusoidal obstructive syndrome
EBV	Epstein–Barr virus
RSV	Respiratory syncytial virus
HMA	Hypomethylating agent
DLI	Donor lymphocyte infusion
